# Characterization of Electrograms from Multipolar Diagnostic Catheters during Atrial Fibrillation

**DOI:** 10.1155/2015/272954

**Published:** 2015-10-25

**Authors:** Prasanth Ganesan, Elizabeth M. Cherry, Arkady M. Pertsov, Behnaz Ghoraani

**Affiliations:** ^1^Biomedical Engineering Department, Rochester Institute of Technology, Rochester, NY 14623, USA; ^2^School of Mathematical Sciences, Rochester Institute of Technology, Rochester, NY 14623, USA; ^3^Department of Pharmacology, SUNY Upstate Medical University, Syracuse, NY 13210, USA

## Abstract

Atrial fibrillation (AF) is the most common arrhythmia in USA with more than 2.3 million people affected annually. Catheter ablation procedure is a method for treatment of AF, which involves 3D electroanatomic mapping of the patient's left atrium (LA) by maneuvering a conventional multipolar diagnostic catheter (MPDC) along the LA endocardial surface after which pulmonary vein (PV) isolation is performed, thus eliminating the AF triggers originating from the PVs. However, it remains unclear how to effectively utilize the information provided by the MPDC to locate the AF-sustaining sites, known as sustained rotor-like activities (RotAs). In this study, we use computer modeling to investigate the variations in the characteristics of the MPDC electrograms, namely, total conduction delay (TCD) and average cycle length (CL), as the MPDC moves towards a RotA source. Subsequently, a study with a human subject was performed in order to verify the predictions of the simulation study. The conclusions from this study may be used to iteratively direct an MPDC towards RotA sources thus allowing the RotAs to be localized for customized and improved AF ablation.

## 1. Introduction

Cardiovascular disease continues to be a major cause of mortality in USA that results in approximately 2,200 deaths a day and an approximate annual cost of $300 billion [1]. Atrial fibrillation (AF), being the most common arrhythmia among all the cardiovascular diseases and a primary cause of stroke [2, 3], results in frequent health care utilization, hospitalizations, and increased risks of heart failure and mortality. Several methods have been developed to treat AF, such as pacemaker surgery and antiarrhythmic drugs. However, an important step forward in the treatment of AF was based on the studies by Haïssaguerre et al. [4], which demonstrated that ectopic beats from the pulmonary veins (PVs) play an important role in triggering AF. This study gave rise to the development of a nonpharmacological ablation therapy called PV isolation, where the atrial tissue in the PV junction is cauterized using radiofrequency energy focused by the ablation catheter. Similar anatomic abalation methods may include additional lesion lines at the roof of the left atrium and along the mitral isthmus. Unfortunately, this procedure has a long-term success rate of only 40% to 60%, primarily because it targets only the abnormal electrical activities originating from PVs and fails to count the sources present outside the PVs.

Previous studies on animal AF have shown that the rotor-like activities (RotAs) that exist outside the PVs contribute to sustaining the AF and hence the ablation procedure should focus on these sites as well [5]. Narayan et al. [6] performed ablation of these sites using unipolar electrograms obtained from a basket catheter, which showed that targeting sites outside the PVs (predominantly the LA region) during ablation provides better results than performing PV isolation exclusively [7]. However, basket is associated with some limitations such as low resolution, bad precision in mapping of anatomical sites, and limited torque capabilities. These limitations can be overcome by using a conventional MPDC instead of a basket catheter and it would still be possible to detect RotAs in humans [8]. Although these studies demonstrate that the RotAs in the LA could be detected, there is no well-defined algorithm to localize them so that the MPDC could be guided towards the RotAs. Unlike PV isolation, where the knowledge of precise location of the AF source is not required because the PV junctions can be anatomically identified and isolated, the cardiac chamber surface is complex and the RotAs cannot be visualized directly. Hence, some knowledge of the location of the RotA source would be beneficial, and it could in turn expedite patient-specific catheter ablation through the use of a RotA localization algorithm.

In recent years, several methods have been developed to determine the location of the RotA source by examining the characteristics of electrograms obtained from catheter. The algorithm developed by Roney et al. [9], which demonstrates a mathematical approach for quantification of the propagation velocity of an action potential and measurement of direction and distance of the RotA using a multipolar catheter, is one such approach to confronting the challenges of localization, along with many other existing mathematical approaches like polynomial surface-fitting algorithms, finite-difference methods, and triangulation techniques [9]. However, detailed information on the variations in characteristics associated with MPDC electrograms, such as conduction delay and cycle length, depending on the distance from the RotA source, is still obscure. If such variations were understood, it is likely that progress in addressing the current challenges in AF, such as achieving improved identification of ablation targets and permanent treatment via AF ablation [10], would be faster. Hence, the major goal of the present study is to investigate the variations in characteristics of the electrograms obtained from the MPDC as the MPDC moves towards the RotA source. The result of this study is expected to facilitate addressing the current challenges in AF ablation.

## 2. Methods

We performed the investigation through a computer simulation, which facilitates positioning a simulated MPDC at any desired location and recording the electrogram. Subsequently, we performed a clinical study in order to confirm the outcome of the simulation study, that is, the inference of the variations in characteristics of the electrograms, as the simulated MPDC moves towards the RotA. A detailed explanation of our study methodology is provided in the following sections.

### 2.1. Simulation

We studied the variations in simulated MPDC electrogram characteristics as the MPDC moves towards the RotA source.

The propagation of activations or action potentials within cardiac tissue involves complex interactions between cellular electrical activity, electrical cell-to-cell communication, and cardiac tissue structure [11]. The action potentials themselves are generated due to transmembrane currents through ion channels, pumps, and exchangers and can be formulated using various mathematical models that are often specific to individual species and specific regions of the heart [12]. Atrial models have been developed for different species such as canine [13] and rabbit [14]. Since our investigation is based on human AF, we considered human atrial models. Previous studies on the simulation of various mathematical models of human atrial cells and tissue have shown that the Cherry and Evans model produces stable spiral waves [12] which serve as a sustained RotA source in our simulation study. Hence, we performed the simulation study by developing a computer simulation of the Nygren et al. human atrial cell model.

#### 2.1.1. Nygren Human Atrial Electrophysiology Model

Nygren et al. [15] published a mathematical model of the human atrial cell myocyte which was based on average voltage-clamp data recorded from the isolated single myocytes. It consists of an assembly of Hodgkin-Huxley-type equivalent circuits for the sarcolemma and a fluid compartment model. This atrial model can reproduce the action potentials produced by the atrial cells that are characterized primarily by ionic currents.

**Figure 1:**
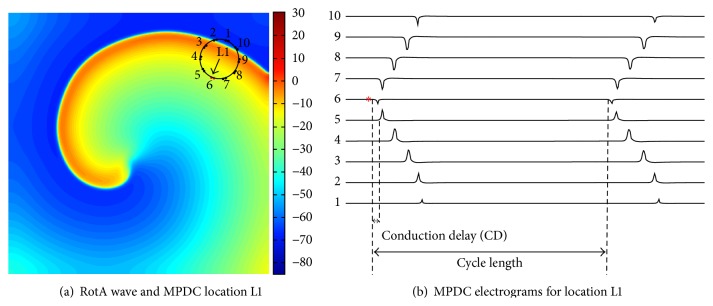
Computer simulation study: (a) RotA wave with the MPDC at location L1 which has the first activated bipole at bipole 6 indicated with the arrow pointing towards it. (b) The electrograms at location L1. The numbers at the left corner indicate the lead number of the corresponding bipole electrode and the asterisk denotes the first activated bipole (FAB). The conduction delay between bipole 5 and the FAB and the cycle length are indicated.

A 10 cm × 10 cm 2D atrial tissue with a spatial resolution of 0.025 cm was simulated using the Nygren atrial cell model. A stable RotA was generated (Figure 1(a)) for a duration of 2 seconds, which led to approximately six rotations. We also implemented a variant of the Nygren et al. atrial model with the following concentrations held constant: [K^+^]_*i*_, [K^+^]_*c*_, [Na^+^]_*i*_, [Na^+^]_*c*_, and [Ca^2+^]_*c*_ [12]. This model creates a larger gyration around a virtual center and a more dynamic meandering at the center of the rotor.

#### 2.1.2. Simulation of MPDC Electrograms

The simulation was programmed to store the output needed to generate pseudo-ECGs (axial current) obtained from specific locations of the 2D tissue (Figure 1(b)). This feature was utilized for simulating an MPDC by providing the program with these location values determined according to the orientation of the electrodes of an MPDC. Using this method, a 20-pole (i.e., 10-bipole) MPDC (replication of Lasso catheter, Biosense Webster, Diamond Bar, CA) with a diameter of 15 mm was simulated and the pseudo-ECG values at those locations were obtained. A detailed description of these MPDC locations and their movement towards the RotA, which constitutes the objective of our simulation study, is provided in the section that discusses the simulation results. The activations of a 10-bipole simulated MPDC are shown in Figure 1(b), where the numbers 1 through 10 indicate the 10-bipole simulated electrodes.

### 2.2. Clinical Study

The primary objective of performing the clinical study is to investigate if the results obtained from the analysis of the variations in the MPDC electrogram characteristics in the simulation studies conform to a practical AF circumstance. This is basically an approach to check the reliability and fidelity of the inference of our simulation study during heterogeneous situations such as AF in a real human atrium. The clinical AF data was collected from a persistent-AF patient (age 59, male) who was undergoing the first AF catheter ablation. The study was approved by the Institutional Review Board at Toronto General Hospital, and the patient provided written informed research consent. Transthoracic echocardiography and transesophageal echocardiography were performed 1 day before ablation in order to evaluate left ventricular ejection fraction and LA size and exclude intra-atrial thrombus. The patient underwent ablation in the postabsorptive state after antiarrhythmic drugs were discontinued for 5 half-lives, with the exception of amiodarone, which was continued. Vascular access was achieved using the femoral vein. A 20-pole catheter (Live-wire, St. Jude Medical, St. Paul, MN) was placed in the coronary sinus. After interatrial septal puncture, a 20-pole variable curve circular mapping MPDC (15–25 mm diameter, 1 mm interelectrode distance, Lasso, Biosense Webster, Diamond Bar, CA) was placed in the LA through a guide sheath. The circular catheter was maintained at maximum diameter whenever possible and maneuvered to the following LA regions in sequence: left PV ostia/antrum, posterior/inferior wall, roof/appendage, right PV ostia/antrum, septum, and anterior wall. At each location, 10 bipolar electrograms (30–500 Hz) were simultaneously recorded for 2.5 seconds at a sampling frequency of 1000 Hz only after catheter stability was achieved.

### 2.3. Signal Processing

#### 2.3.1. Preprocessing of MPDC Electrograms

The preprocessing of the simulated and clinically obtained MPDC electrograms was performed separately using custom software written in MATLAB as explained below.

*Simulated Electrograms*. The activation times of the simulated MPDC electrograms were first annotated at the locations of the maximum negative deflections [16, 17].

*Clinically Obtained Electrograms.* After the ablation procedure was completed, the analysis of clinical data was performed offline. MPDC recordings were excluded if positional stability was poor during the recording or if there are some missing bipoles. The data that is required by the developed algorithm is readily available in the existing electroanatomical mapping systems. The* xyz* coordinates of each bipole as well as the 3D structure of the left atrium were obtained from the clinical mapping system so identification of the unstable catheter recordings, catheter deformities, or poor contact bipoles was easily done in our custom program. Local activation of the remaining bipolar electrograms was defined automatically from the maximum peak positive or negative deflection with reference to the isoelectric baseline after which far-field ventricular activation was identified and excluded from the analysis of local activation. The electrograms obtained from the various locations of recording in the LA were first investigated by a clinical electrophysiologist to look for RotA sources. A RotA was clinically defined if the time interval between consecutive local activations around the circular catheter encompassed the local AF cycle length such that the head of the advancing wavefront met the tail of the subsequent wavefront [8]. To ensure that the arrhythmias analyzed were stable, only the MPDC recordings with electrograms consisting of ≥3 cycles of similar activation pattern and bipolar electrogram morphology were retained for further analysis.

#### 2.3.2. Characterization of MPDC Electrograms

In order to accomplish the goal of our study, which is to investigate the variations in the characteristics of MPDC electrograms, the following three characteristics were determined at each recording site: (1) first activated bipole (FAB), (2) total conduction delay (TCD), and (3) average cycle length (CL) at the FAB.

FAB was determined as the first bipole that encounters the wavefront (i.e, the bipole with the earliest activation time (AT)). The AT of each bipole is calculated with respect to the beginning of the recordings. For example, in Figure 1(b), the FAB denoted by the asterisk implies that at location L1 the earliest activation occurred at bipole electrode number 6 (bipole 6) of the MPDC. The FAB is shown as the head of the arrow in Figure 1(a).

The conduction delay (CD) of a particular bipole is calculated as the interval from each local activation to that of the neighboring bipole with respect to its FAB. For example, in Figure 1(b), the conduction delay of bipole 5 (CD_5_) is calculated as the time interval between the activations of bipole 6 (i.e., FAB) and bipole 5, as illustrated in the figure. CD_4_ will be calculated with respect to bipole 5 and so forth as follows:

(1) $$\begin{array}{c} \text{C}{\text{D}}_{i}=\left|\text{A}{\text{T}}_{i}-\text{A}{\text{T}}_{i+1}\right|\\ i=\text{FAB}-1\;\text{to}\;\;1. \end{array}$$

The calculation of CD_6_ to CD_9_ will be continued with respect to the subsequent bipole as shown as follows:

(2) CDi=ATi+1−ATii=FAB to  9.

Additionally, CD_10_ is calculated as the time interval between bipole 1 and bipole 10 as given as follows:

(3) $$\begin{array}{c} \text{C}{\text{D}}_{10}=\left|\text{A}{\text{T}}_{1}-\text{A}{\text{T}}_{10}\right|. \end{array}$$

**Figure 2:**
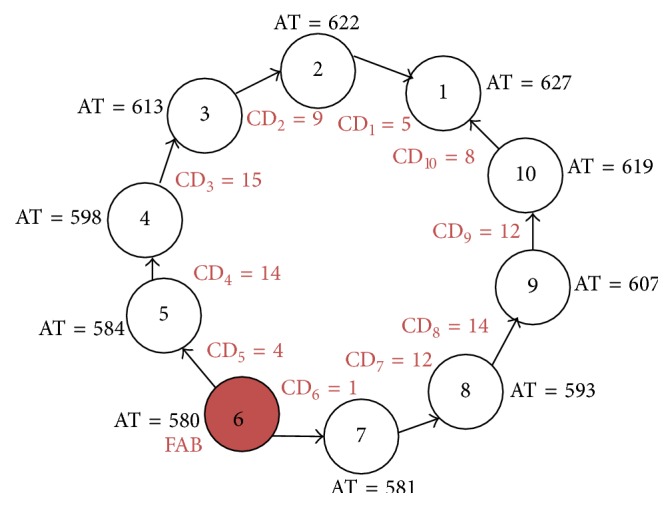
This diagram illustrates the FAB and TCD calculations for the electrograms recorded using an MPDC. Bipole 6 is the FAB and TCD is 94 ms, which is the sum of CD_1_ to CD_10_. AT: activation time. CD: conduction delay. All times are given in ms.

Figure 2 illustrates how our CD and TCD calculations are performed for the MPDC in Figure 1(b). Bipole 6 is the FAB with the earliest activated time of 580 ms. CD_1_ to CD_10_ are calculated as explained above and shown for each bipole. This procedure is continued for all cycles of the electrogram and the CD for every bipole electrode is averaged over the number of cycles to obtain a single value of CD for each electrode. Finally, the summation of the average CD of every electrode is calculated to obtain the TCD at each recording site. The mathematical expression for TCD at any MPDC location *l* is given by

(4) $$\begin{array}{c} \text{TC}{\text{D}}_{l}=\overset{10}{\underset{i=1}{\sum }}\text{C}{\text{D}}_{i}. \end{array}$$

The cycle length is the time delay between two successive activations in the same electrode during consecutive cycles (Figure 1(b)). Here, we calculate CL using only the FAB to have a single CL for a given MPDC location. The cycle length of the FAB is calculated for different cycles and averaged over the number of cycles to obtain an average cycle length (i.e., CL) for the FAB.

## 3. Results and Discussion

### 3.1. Simulation Study

**Figure 3:**
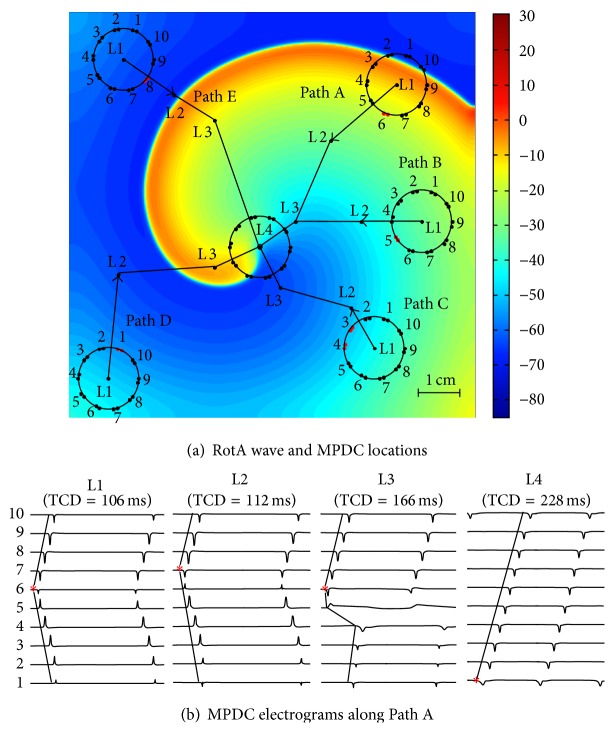
Simulation results: (a) L4 is the location of the known RotA source. The circles show the MPDC at the start point (L1) of each of Paths A to E; in every step of each path, the MPDC moves forward towards the RotA source; (b) the electrograms show the conduction pattern of path A from L1 to L4. The TCD increases from 106 ms to 228 ms, as reported on the top of each MPDC electrogram. The numbers at the left corner indicate the lead number of the corresponding bipole electrode of the MPDC.

Figure 3 illustrates the simulation study. For investigating the variations in characteristics of MPDC electrograms as the MPDC moves towards a RotA source (L4 in Figure 3), the locations of the MPDC recordings in the simulation were selected as shown in Figure 3. Initially we started by selecting a random location farther from the RotA (i.e., L1). The MPDC was then advanced to a random location towards the direction of the RotA source (i.e., L2) as assessed visually. This procedure was repeated to L3 and then L4 until the MPDC reached the RotA source (i.e., L4). This guidance procedure was repeated starting the MPDC from 5 different locations forming 5 paths (Path A to Path E in Figure 3(a)). The MPDC electrograms obtained from Path A are shown in Figure 3(b). We calculated the FAB, TCD, and CL at each location using the procedure discussed previously in Section 2.

**Figure 4:**
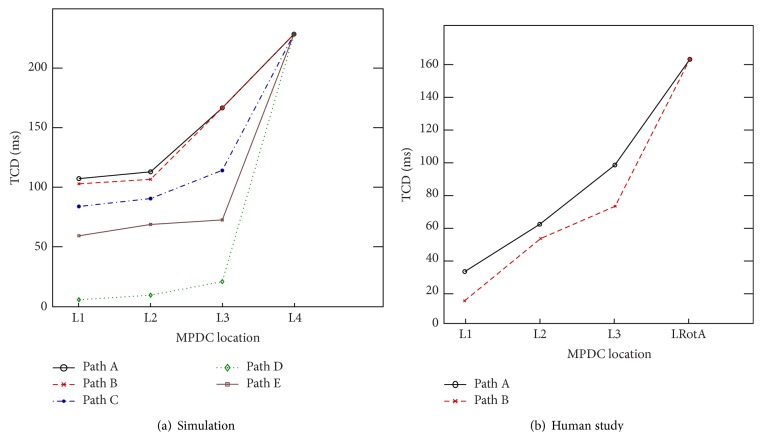
Variation of total conduction delay. Variation in TCD with respect to the MPDC location: (a) In simulation study, the TCD shows an increasing gradient as the MPDC moves from L1 towards the RotA at L4, in all the paths. (b) The plot demonstrates the increasing gradient of TCD in the clinical study, in both Path A and Path B when the MPDC moves from L1 to LRotA, with the location of every successive step directed towards the first activated bipole of the current location.

**Figure 5:**
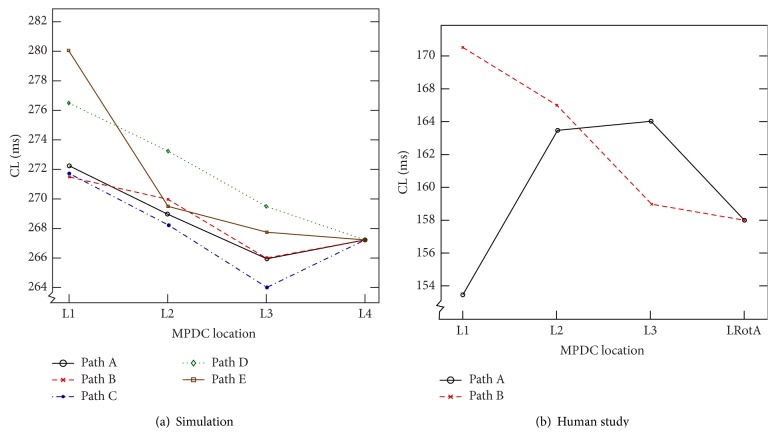
Variation of average cycle length. Variation in average cycle length (CL) of the FAB with respect to the MPDC location: (a) In simulation study, the CL of Path D and Path E shows a decreasing gradient as the MPDC moves from L1 towards the RotA at L4. However, this decreasing gradient is not consistent in the other paths, where the CL decreases as the MPDC moves from L1 to L3 but increases at L4. (b) The plot demonstrates the inconsistent CL behavior observed in the clinical study. In Path A, the CL shows a decreasing gradient when the MPDC moves from L1 to LRotA, with the location of every successive step directed towards the FAB of the current location; however in Path B, the CL gradient is inconsistent; it increases as the MPDC moves from L1 to L3 with the location of every successive step directed towards the FAB of the current location, but it decreases at the location of RotA (i.e., LRotA).

While moving the MPDC towards the RotA source (moving from L1 to L2, L3, and L4) and determining the electrogram characteristics at each location along the movement path, the variations in TCD and CL were examined.  Figure 4(a) shows the TCD values of the five paths as the MPDC was moved from L1 to the RotA source at L4. As can be seen in this figure, TCD increased along all the paths. For example, as can be seen in Figure 3(b) the TCD value increased from 106 ms to 112 ms, 166 ms, and 228 ms as the MPDC moved from L1 to L2, L3, and L4 (the RotA location). We also observed that, at each location, the FAB was the bipole closest to the RotA source. Additionally, we generally observed a decreasing pattern in the CL of the FAB as the MPDC moved towards the RotA; however, the pattern was not consistent in some cases, as shown in Figure 5(a). We repeated the experiment using the modified Nygren model as explained in Section 2.1. The steady-state spiral wave trajectories found using this model created a larger gyration on a circle with a radius of greater than 1 cm. Our calculation resulted in a similar behavior showing that the TCD increased along all the paths even in the presence of a meandering rotor. These observations from the simulation study led to the following predictions:

- Moving the MPDC in the direction of the FAB with respect to the center of the catheter will guide the MPDC towards the RotA source.
- A* decreasing* (increasing) TCD as the MPDC moves from one location to a neighboring location indicates that the MPDC is moving* away from* (towards) the RotA source.

#### 3.1.1. Clinical Study

**Figure 6:**
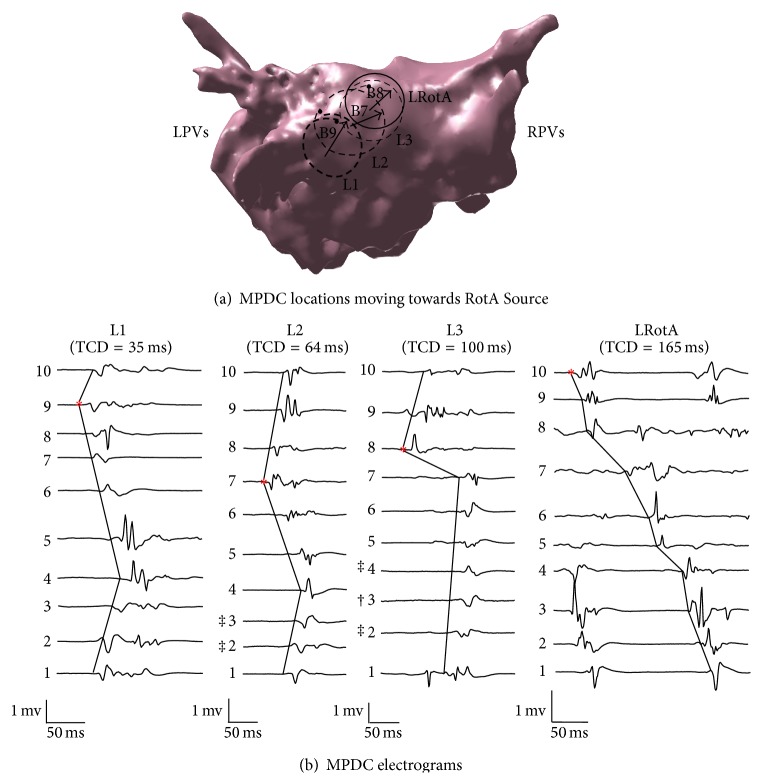
Clinical study, Path A: (a) The dashed rings (L1, L2, and L3) show the location of MPDCs moving towards the RotA which is represented by a solid ring (LRotA); the direction of the first activated bipole is indicated in the figure with arrows and is B9, B7, and B8 for L1, L2, and L3, respectively. The filled circle represents bipole 1 of each MPDC. (b) A single cycle from the electrograms obtained at each of L1, L2, and L3 and LRotA are shown along with the corresponding TCD reported at the top of each electrogram and the first activated bipoles are indicated by the asterisks. The bipole electrode numbers 1 to 10 corresponding to each lead are indicated beside every bipole. RPVs: right pulmonary veins; LPVs: left pulmonary veins. † Voltage scaling decreased 4×. ‡ Voltage scaling decreased 2×.

**Figure 7:**
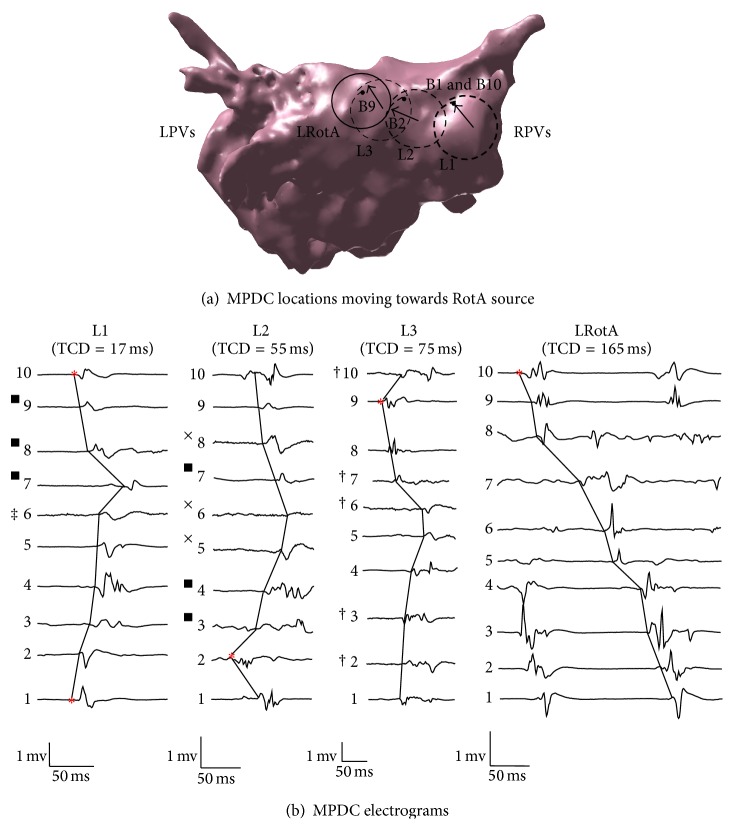
Clinical study, Path B: (a) The dashed rings (L1, L2 and L3) are the location of MPDCs moving towards the RotA represented by a solid ring (LRotA); the first activated bipole is denoted by the corresponding bipole number and an arrow pointing to it. The solid ring indicates the location of the MPDC recording that encompasses the rotor source. ∗ represents bipole 1 of each MPDC. (b) A single cycle from the electrograms obtained at each of L1, L2, L3, and LRotA is shown along with the corresponding TCD reported at the top of each electrogram; the first activated bipole is indicated by the asterisks; the corresponding bipole electrode numbers 1 through 10 are indicated beside every bipole. RPVs: right pulmonary veins; LPVs: left pulmonary veins. ■ Voltage scaling decreased 2×. † Voltage scaling decreased 4×. ‡ Voltage scaling decreased 5×. × Voltage scaling decreased 6×.

One RotA source was located along the posterior wall as confirmed by the clinical electrophysiologist (LRotA in Figures 6(a) and 7(a)). The RotA was reported for 0.5 ms, leading to 4 rotations. Our objective was to use the MPDC electrogram characteristics and verify if the predictions of the simulation study (i.e., the increasing TCD and the direction of FAB as the MPDC moves towards the RotA source) can be verified in a clinical AF case.

In order to accomplish this objective, from the locations of MPDC recordings, we selected a random location and looked for an immediate neighboring location that satisfied the following criteria: (1) the MPDC location was in the direction given by the FAB of the current location, and (2) the TCD at this location was greater than that of the current location. We continued following such locations successively until we could not find a neighboring MPDC location with such criteria or we reached the RotA source. In cases where we could not find a neighboring MPDC location with our criteria, we started with any of the neighboring locations and reset the algorithm until we found a location that satisfied the criteria. We repeated this exhaustive procedure for all the MPDC locations that were recorded in the clinical study and found all the paths that consisted of successive MPDC locations and started from one location farther from the RotA source and ended up in the location of the RotA source, thus forming a path always directed towards the RotA. Four such paths were found; the two longest paths along with their electrograms are shown in Figures 6 and 7. In these figures, each arrow indicates the direction of the FAB of its corresponding MPDC and the TCD is calculated with respect to the FAB. For the path shown in Figure 6(a), it can be seen that the value of the TCD represents an increasing pattern which is from 35 ms through 64 ms to 100 ms until the MPDC converges on the RotA where the TCD is observed to be 165 ms. The path shown in Figure 7 presents the same behavior. The overall TCD increases for all the four paths from the clinical study as shown in Figure 4(b). However, the CL corresponding to the FAB of these four paths did not show consistent variations, similar to our simulation study (Figure 5(b)). Our observations suggest that the results obtained from our simulation study are valid for a case of cinical human AF as well, thereby suggesting that when the MPDC was at a location far from the RotA, it could have been guided to the location of the RotA source by monitoring the FAB and the variations in TCD.

In addition to calculating the TCD and CL characteristics, we studied the relationship between TCD and CL when the MPDC is exactly converging on the RotA source (i.e., L4). At this location, the MPDC encircles the RotA source and any cycle of the RotA wave is completed at the same MPDC electrode where the cycle started. Hence, the TCD (i.e., TCD_RotA_) is almost equal to the CL (i.e., CL_RotA_): TCD_RotA_≃CL_RotA_.

#### 3.1.2. Clinical Implications

Current AF ablation procedures construct the 3D electroanatomic mapping of the LA by maneuvering a conventional MPDC along the LA endocardial surface. However, the procedures are limited to pulmonary vein isolation and other linear ablation (i.e., roof and mitral isthmus) performed on various regions of the LA where the regions are decided based on the atrial anatomy. Hence it remains unclear how to utilize the information provided by the MPDC to locate RotAs. Based on our previous study, which supports the ability of an MPDC to detect sustained RotAs in human AF [8], we believe that utilizing the information recorded during MPDC movements can significantly improve AF target detection over the current practice, while remaining economically attractive and safe for patients (no basket catheters). The results from our simulation and human AF study suggest that it is possible to guide an MPDC towards a RotA source by monitoring the characteristics of the MPDC recordings. Previous electrogram characterization studies mainly use a single bipole to characterize the electrograms, such as CL or dominant frequency from the time or frequency domain to look for the RotAs. In this study, we investigated the collective information from a 10-bipole MPDC to determine a technique to guide a catheter towards the RotA source using the FAB and TCD. Therefore, the results of both simulation and clinical studies demonstrate that moving an MPDC in the direction of the FAB while an increasing gradient in the TCD is observed could potentially guide the catheter towards the RotA source.

#### 3.1.3. Study Limitations

Only a small number of all possible paths to the RotA that could have been formed were used in the simulation study. Hence, it is possible that other MPDC electrogram characteristics could exist that were not observed in this study. The clinical AF study was limited to offline analysis of the recorded MPDC sites. Hence, there could be multiple simultaneous RotA sources, but only one was recorded during the 3D electroanatomy mapping. This study can be further improved by performing a real-time data analysis during the 3D electroanatomy mapping. Additional clinical study can also be performed in future in order to consider common variations in persistent AF. The simulation study can also be further developed by simulating multiple RotA sources in the human atrial anatomy in order to investigate the changes to the MPDC characteristics in the presence of multiple RotAs and also possibly ectopic sources in order to replicate the effect of the ectopic beats from PVs.

## 4. Conclusion

In this study, we investigated the variations in characteristics such as the TCD and CL from the MPDC electrograms as the MPDC moved towards a RotA source. We used two approaches for the investigation: a computer simulation study was developed using an existing well-known mathematical model of human atrial cell and the second approach was performing studies with a human subject. As a result of the simulation study, we observed a consistent increase in the TCD as the MPDC moved towards a RotA source. We also observed that the MPDC electrode that encounters the earliest activation was always directed towards the RotA source. In a clinical AF case, the phenomenon of the increasing gradient of TCD and the significance of the direction of FAB, if followed, have the potential to guide the MPDC to the location of RotA source; however, we did not observe any similar consistent pattern from the CL. Our findings may be used to iteratively direct an MPDC towards RotAs and allow the RotAs to be successfully localized for customized and improved AF ablation.
